# Country development and manuscript selection bias: a review of published studies

**DOI:** 10.1186/1471-2288-6-37

**Published:** 2006-08-01

**Authors:** Reza Yousefi-Nooraie, Behnam Shakiba, Soroush Mortaz-Hejri

**Affiliations:** 1 Center of Evidence Based Medicine, Tehran University of Medical Sciences, Shariati hospital, Kargar shomali Ave, Tehran, Iran; 2 Students' Scientific Research Center, Third floor, Faculty of medicine, Tehran University of Medical Sciences, Tehran, Iran

## Abstract

**Background:**

Manuscript selection bias is the selective publication of manuscripts based on study characteristics other than quality indicators. One reason may be a perceived editorial bias against the researches from less-developed world. We aimed to compare the methodological quality and statistical appeal of trials from countries with different development status and to determine their association with the journal impact factors and language of publication.

**Methods:**

*Selection criteria*: Based on the World Bank income criteria countries were divided into four groups. All records of clinical trials conducted in each income group during 1993 and 2003 were included if they contained abstract and study sample size. *Data sources*: Cochrane Controlled Trials Register was searched and 50 articles selected from each income group using a systematic random sampling method in years 1993 and 2003 separately. *Data extraction*: Data were extracted by two reviewers on the language of publication, use of randomization, blinding, intention to treat analysis, study sample size and statistical significance. Disagreement was dealt with by consensus. Journal impact factors were obtained from the institute for scientific information.

**Results:**

Four hundred records were explored. Country income had an inverse linear association with the presence of randomization (chi2 for trend = 5.6, p = 0.02) and a direct association with the use of blinding (chi2 for trend = 6.9, p = 0.008); although in low income countries the probability of blinding was increased from 36% in 1993 to 46% in 2003. In 1993 the results of 68% of high income trials and 64.7% of other groups were statistically significant; but in 2003 they were 66% and 82% respectively. Study sample size and income were the only significant predictors of journal impact factor.

**Conclusion:**

The impact of country development on manuscript selection bias is considerable and may be increasing over time. It seems that one reason may be more stringent implementation of the guidelines for improving the reporting quality of trials on developing world researchers. Another reason may be the presumptions of the researchers from developing world about the editorial bias against their nationality.

## Background

Publication bias is defined as "the tendency to publish research results based on the strength and direction of findings"[1]. It is well documented that studies with non-significant or negative results are substantially less likely to be submitted for publication[2,3]. In addition, the proportion of published articles from low income countries is lower than more developed nations in many research fields, including psychiatry[4], cardiovascular disease[5] and epidemiology[6]. A recent survey concluded that researchers from less-developed countries believe that one likely reason is a substantial editorial bias against their work[7]. The tendency of editors to publish research results based on study characteristics other than quality indicators is called generally manuscript selection bias in the current study, which is broader than the publication bias definition. The purpose of present study was to compare the methodological quality and statistical appeal of published trials from countries with different developmental status and to determine their association with the journal impact factor and language of publication.

## Methods

### Selection criteria

Based on the World Bank income criteria countries were divided into low, lower-middle, upper-middle and high income groups[8]. All records of clinical trials conducted in each income group during 1993 and 2003 were included if they contained abstract and the number of study participants (study sample size) could be calculated from the abstract content.

### Search strategy

Cochrane Central Register of Controlled Trials (2005, issue1) was searched for all trials including the names of countries in each income group in the Institution field of the records. Fifty articles in each income group were selected using a systematic random sampling method in years 1993 and 2003 separately, which comprised of the total of 400 citations. All the citations identified by the above searches were screened and animal studies, non interventional studies or conference abstracts were excluded by consensus; then the sampling was performed once more to replace the excluded citations.

### Data extraction

Data were extracted by two reviewers on the language of publication, use of randomization, blinding (masking, placebo or sham), intent(ion) to treat analysis, study sample size and statistical significance. An article was classified as non-English if it was described in the bibliographic details. Quality assessment was restricted to the statement of randomization, blinding and intention to treat analysis. The results of a trial was called significant when there was at least a p < 0.05, the 95% confidence interval excluded no effect, or the statistical significance was stated qualitatively in the results section for any finding of the study. Disagreement was dealt with by discussion. Information on journal impact factor was obtained from the 1994 and 2003 editions of the Science Citation Index Journal Citation Reports.

### Statistical analysis

The Chi square for trend test was applied to the cross-tab of randomization (or blinding, significance) and country income to assess the differences between levels of country income.

Forward stepwise linear regression was performed to model the relation of country income to journal impact factor. We included the following potential confounders as covariates: language of publication, study sample size, randomization, blinding, statistical significance and publication year.

As the journal impact factor was not normally distributed, we performed logarithmic transformation. We dichotomized the country income (High income = 1, others = 0), publication year (2003 = 1, 1993 = 0), language of publication (English = 1, others = 0), use of randomization, use of blinding and statistical significance. All analyses were undertaken in SPSS version 11.5 (SPSS, Inc., Chicago, IL). A p value of less than 0.01 was considered statistically significant.

## Results

Four hundred records were explored. There were 377(94.3%) English language articles. According to the title, abstract or keywords the number of Controlled clinical trials which refer to the studies that compare one or more intervention groups to one or more comparison (control) groups was 384(96%). The number of randomized controlled trials was 298(74.5%), before and after studies was 14(3.5%) and the frequency of crossed-over studies was 39(9.7%). Double-blind and single-blind methods were mentioned in 141(35.3%) and 39(9.8%) of records respectively. The loss to follow-up percent was stated in 29 abstracts and 5 studies reported an intention-to-treat analysis.

### Country income

In 1993 the results of 68% of high income trials and 64.7% of other groups were statistically significant; but in 2003 they were 66% and 82% respectively (table 1). Whilst in low income countries the probability of blinding was increased from 36% in 1993 to 46% in 2003, in high income countries there was about fourteen percent decrease. Country income had a non-significant inverse association with the presence of randomization (Chi square for trend = 5.6, p = 0.02) and a direct significant association with the use of blinding (Chi square for trend = 6.9, p = 0.008). As shown in table 2 in 1993 the articles from high income countries had an odds ratio of 1.17 for having statistically significant results compared with the other countries, after adjustment for the language of publication. The odds ratio decreased to 0.41 in the year 2003. There were 2 studies in high-middle income group and 3 in low-middle income group reported an intention to treat analysis.

**Table 1:** The frequency of randomized studies, use of blinding and statistical significance among different income groups in the years1993 and 2003 (numbers in the parentheses represent the 95% CIs of the absolute change)

	**Low**	**Lower-middle**	**Upper-middle**	**High**
**Randomized studies:**				
1993	38/50(76%)	36/50(72%)	34/50(68%)	31/50(62%)
2003	44/50(88%)	38/50(76%)	39/50(78%)	37/50(74%)
Absolute change (%)	12 (-2 to 27)	4(-13 to 21)	10(-7 to 27)	12(-6 to 30)

**Blinded studies:**				
1993	18/50(36%)	23/50(46%)	25/50(50%)	30/50(60%)
2003	23/50(46%)	16/50(32%)	22/50(44%)	23/50(46%)
Absolute change (%)	10(-9 to 29)	-14(-32 to 5)	-6(-25 to 14)	-14(-33 to 5)

**Significant results:**				
1993	29/50(58%)	30/50(60%)	38/50(76%)	34/50(68%)
2003	40/50(80%)	41/50(82%)	42/50(84%)	33/50(66%)
Absolute change (%)	22(4 to 39)	22(5 to 39)	8(-7 to 24)	-2(-20 to 16)

**Table 2:** The odds ratios of the presence of randomization, blinding and reporting significant results in the articles from high income countries to the reports from the other nations adjusted for the language of publication

	**Adjusted OR(95% CI)**
**Randomization:**	
1993	0.59(0.29 to 1.17)
2003	0.64(0.29 to 1.36)

**Blinding:**	
1993	1.93(0.99 to 3.74)
2003	2.36(1.08 to 5.13)

**Statistical Significance:**	
1993	1.17(0.58 to 2.34)
2003	0.41(0.20 to 0.85)

### Journal impact factor

In forward stepwise linear regression model, fitted on the log impact factor the country income, the language of publication, study sample size and the publication year were independent predictors of journal impact factor which remained in the model (table 3). The final model accounted for 14.7% of the variance in journal impact factor and was statistically significant (F = 12.2, p < .0001).

**Table 3:** Linear regression model fitted on log impact factor

Explanatory variables*	**B coefficient(95% CI)**
Country income	0.490(0.249 to 0.730)
Publication year	0.269(0.060 to 0.479)
Log Study sample size^†^	0.197(0.103 to 0.291)
Language of publication	0.976(0.247 to 1.70)
Randomization	Not selected
Blinding	Not selected
Statistical significance	Not selected

*The randomization, use of blinding and statistical significance were not selected in the final forward stepwise regression model.

^†^*Logarithmic transformation because of a large right skewness*.

## Discussion and conclusion

The frequencies of blinding and statistical appealing indicators in the published articles from low income nations have increased more than developed countries during the past ten years and the presence of randomization had a negative association with country development in both occasions. On the one hand, this finding may be due to an enhancement of exclusive and biased use of critical appraisal checklists by the editors of western medical journals. On the other hand, it may refer to the developing world authors, who might be more keen to selectively report the studies that are larger, have less serious limitations, and containing positive and significant findings in international english language journals, because of the presumption that editors and reviewers are biased against their nationality[9,10].

The poorer countries are underrepresented both on the editorial boards and in the pages of medical journals, which stated as a systematic bias against the diseases of poverty [11].

Some aspects of this bias is demonstrated in the studies, that the majority of the editorial board members of international medical journals come from nations with a high human development index [12,13] and the studies enrolling some participants from the United States are more likely to be published[14]. In our study the effect of country development on the odds of the statistical significance of published studies was considerable after adjustment for the language.

We didn't find a significant relationship between methodological quality indicators of the research and the impact factor of the medical journal that cited it. Conversely, study sample size and country income were more important predictors of journal impact factor, despite the generally accepted belief that the journal impact factor could be used as a measure of journal quality[15,16].

Consequently we can interpret the result as an alarm sign that the impact of country development on the manuscript selection bias is increasing by time. Publication bias is a preventable problem[17] and we suggest that "Developing country bias" should be intentionally stated as an independent factor which can contribute to biased inclusion of research manuscripts for publication; Publication bias awareness raising programs should be planned for developing world researchers; In addition, current standards for reporting trials should be enforced more equally on the articles from different nations [18].

We used The Cochrane Central Register of Controlled Trials (CENTRAL) as the source of studies which serves as the most comprehensive source of records related to controlled trials. As of January 2003, CENTRAL contained just over 350,000 citations to reports of trials and other studies potentially relevant to Cochrane Reviews. CENTRAL includes citations to reports of controlled trials that might not indexed in MEDLINE, EMBASE or other bibliographic databases; citations published in many languages; and other sources that are difficult to access[19].

Present study has several limitations. First, the main problem with our study is the lack of a denominator i.e. the study presents no data on the total number of papers submitted only on those papers published. If only very small numbers of papers are published then this introduces a bias so that it is not possible to make assumptions about the quality of papers as a whole. Second, all assessments are done based on the data provided in title, abstract and keywords and obviously several useful information might be omitted. Abstracts from higher impact journals might contain more information and therefore yield higher quality scores than those from other types of journal. If so, the real differences between low and high income countries may be more than what is reported in our study. Poor (or even fraudulent) documentation and adoption of CONSORT statement from journals may affect the results. Another important constraint of relying on the abstract is that the failure to report a feature, e.g. blinding in the abstract does not necessarily equate with failure to carry it out. Third, we limit our investigation to clinical trials and may underestimate the real heterogeneity among different income groups. Fourth, data extractors were not blinded to country and journal. We reduced its effects by considering the most objective characteristics of the studies that could be extracted by the abstracts. Fifth, the country income has been presumed as a surrogate for developmental status. Finally, all studies included in the present study were cited in Cochrane Central Register of Controlled Trials which serves as the most comprehensive source of records related to controlled trials, but mainly consists of the articles indexed in MEDLINE and EMBASE. There is a proven language bias in these databases[20] and may limit the external validity of our results.

Because of the above mentioned drawbacks we suggest to interpret the results of the present study with more caution.

## Competing interests

The author(s) declare that they have no competing interests.

## Authors' contributions

*Study concept and design*: Yousefi-Nooraie. *Acquisition of data*: Yousefi-Nooraie, Mortaz-Hejri. *Analysis and interpretation of data*: Yousefi Nooraie, Shakiba, Mortaz-Hejri. *Drafting of the manuscript*: Shakiba, Yousefi-Nooraie. *Critical revision of the manuscript for important intellectual content*: Yousefi-Nooraie, Shakiba.

## Pre-publication history

The pre-publication history for this paper can be accessed here:


